# The effect of smoking on the outcomes of arthroscopic microfracture for osteochondral lesions of the talus

**DOI:** 10.1371/journal.pone.0321894

**Published:** 2025-04-22

**Authors:** Gun-Woo Lee, Min-Su Lee, Jong-Eun Kim, Keun-Bae Lee

**Affiliations:** 1 Department of Orthopedic Surgery, Chonnam National University Hospital, Gwangju, Republic of Korea; 2 Department of Orthopedic Surgery, Chonnam National University Medical School, Gwangju, Republic of Korea; Far Eastern Memorial Hospital, TAIWAN

## Abstract

**Background:**

Smoking is known to negatively affect the outcomes of orthopedic procedures, but its impact on arthroscopic microfracture for osteochondral lesions of the talus (OLT) remains unclear. We aimed to compare clinical outcomes and the status of repaired cartilage following arthroscopic microfracture for small to medium-sized OLT in smokers versus nonsmokers.

**Methods:**

We enrolled 239 patients (250 ankles), dividing them into smoker (56 patients, 59 ankles) and nonsmoker groups (183 patients, 191 ankles). The primary outcome measure was the FAOS (Foot and Ankle Outcome Score). The AOFAS (American Orthopaedic Foot & Ankle Society) ankle-hindfoot scale, SF-36 PCS (Short Form-36 Physical Component Summary) score, and VAS (Visual Analog Scale) for pain were included as secondary outcomes. Preoperative magnetic resonance imaging (MRI) assessed lesion size, location, and subchondral cyst presence. Postoperative cartilage repair status was evaluated using the Magnetic Resonance Observation of Cartilage Repair Tissue (MOCART) score on 3.0-T MRI.

**Results:**

The mean OLT sizes were 74.4 mm^2^ in smokers and 69.9 mm^2^ in nonsmokers on preoperative MRI. The mean age was 35.9 years in smoker group and 38.8 years in nonsmoker group (p = 0.157). The overall mean follow-up duration was 83.6 months (range, 24–217), with no significant intergroup difference (p = 0.582). There was no significant difference in primary and secondary clinical outcome variables between the two groups at the final follow-up (p > 0.05). In terms of postoperative MRI, 75 ankles (18 smokers, 57 nonsmokers) assessed repaired cartilage status and the mean total MOCART score was significantly lower in smokers (65.0, range 30.0–85.0) compared with nonsmokers (73.7, range 40.0–95.0; p = 0.027). Particularly, the smoker group had significantly lower MOCART scores for surface for repair tissue and signal intensity of the repair tissue variables, respectively (p = 0.019, p = 0.008).

**Conclusion:**

Although smoker group showed worse status of repaired cartilage on postoperative MRI, the smoker group reported comparable clinical outcomes to those of the nonsmoker group following arthroscopic microfracture for small to medium-sized OLT over a mean follow-up of 7 years. However, caution should be taken in interpreting our conclusion and further larger studies are needed for robust conclusions.

## Introduction

Surgical interventions for osteochondral lesions of the talus (OLT) include joint-preserving options like bone marrow stimulation (BMS), fragment fixation, subchondroplasty, and tissue transplantation.[1–5] Arthroscopic BMS via microfracture or drilling is the most common first-line treatment for small to medium-sized primary OLT due to its simplicity, effectiveness, and low morbidity.[6,7] This technique involves releasing mesenchymal stem cells by penetrating the subchondral bone, leading to fibrocartilage formation.[1,8,9] The long-term efficacy of BMS has been questioned due to fibrocartilage’s biological inferiority and the incomplete healing of subchondral bone.[10,11] However, recent studies reported satisfactory functional outcomes following arthroscopic BMS over intermediate to long-term follow-up.[6,12] In addition, recent meta-analysis has shown that none of the treatment options showed any superiority over others for primary OLT.[7]

The outcomes of microfracture, a type of BMS, are influenced by factors such as patient age, body mass index (BMI), smoking status, lesion size, containment type, morphology of the ankle, and bone marrow edema.[6,12–14] In general, smoking negatively affects cartilage healing by increasing platelet aggregation, decreasing microvascular prostacyclin levels, and impairing fibroblasts, red blood cells, and macrophages.[15,16] Previous studies have shown that smokers have worse postoperative outcomes and higher complication rates after autologous matrix-induced chondrogenesis (AMIC) for OLT.[13,17] Additionally, smokers show less clinical improvement and are less likely to form hyaline-like cartilage compared with nonsmokers after autologous chondrocyte implantation (ACI) for knee chondral defects.[18] The impact of smoking on arthroscopic microfracture outcomes for OLT, however, is not well understood.

We hypothesized that smoking leads to poorer clinical outcomes and cartilage repair following arthroscopic microfracture in OLT patients. Therefore, we aimed to compare clinical outcomes and repaired cartilage status after arthroscopic microfracture for small to medium-sized OLT in smokers versus nonsmokers.

## Materials and methods

### Patients

This study was approved by our hospital’s institutional review board (CNUH-2021–228), and informed written consent was obtained from all patients. We retrospectively reviewed a consecutive series of 322 patients (391 ankles) underwent arthroscopic microfracture for OLT from January 2005 through December 2021. Indications for microfracture included symptomatic full-thickness chondral or osteochondral lesions unresponsive to nonoperative management for at least 6 months. Inclusion criteria were primary surgery, lesion size under 150 mm^2^, at least 24 months of follow-up, and known smoking status. Smokers were defined as individuals who had smoked over 100 cigarettes in their lifetimes at the time of surgery and continued smoking for at least 24 months after primary surgery.[19] Conversely, nonsmokers were defined as those who had either never smoked or had quit smoking more than 10 years before surgery. We excluded patients with any prior or concurrent ankle operations not related to OLT (e.g., fracture, instability, deformity correction), rheumatoid arthritis, ankle osteoarthritis of Kellgren-Lawrence grade 2 or higher, and age over 65 years. Additionally, patients who did not clearly meet the criteria for either the smoker or nonsmoker group were excluded. Ultimately, 239 patients (250 ankles) were enrolled and categorized into smoker (56 patients, 59 ankles) and nonsmoker groups (183 patients, 191 ankles). All procedures were performed by 2 orthopedic foot and ankle surgeons at the same institution.

### Surgical techniques and rehabilitation

Arthroscopic microfracture was performed under general or spinal anesthesia with a thigh tourniquet applied. A 2.5-mm, 30° arthroscope (Linvatec, Largo, FL, USA) facilitated the procedure through 3 portals (anteromedial, anterolateral, and posterolateral). The posterolateral portal was occasionally used for better access and visualization in a few cases where lesions extended far posteriorly, with the patient in the supine position and the ankle positioned at the edge of the operating table. Unstable cartilage and small osteochondral fragments were excised, and lesion margins were squared off with a curette for blood clot adherence. Microfracture awls created penetrations in the subchondral bone, spaced 3–4 mm apart and deep, around the lesion’s periphery.

Postoperatively, ankles were immobilized in a neutral position with a posterior splint for 2 weeks. Patients commenced tolerable weight-bearing in a walking boot, alongside active ankle motion and strengthening exercises 2 weeks after surgery. Full weight-bearing without support was permitted at 6–8 weeks, with a return to sports activities allowed between 4–6 months postoperatively.

### Arthroscopic evaluation

Primary arthroscopic findings were evaluated and classified using the Ferkel and Cheng (F–C) staging system for the comparison of baseline characteristics of the two groups.[20] Two observers assessed all arthroscopic findings, and in cases of disagreement, the less favorable assessment prevailed.

### Clinical evaluation

Clinical assessments were conducted preoperatively and at final follow-up. The primary outcome was the Foot and Ankle Outcome Score (FAOS),[21] a subjective patient-reported measure with established validity and reliability in OLT patients.[22] Secondary outcomes included the AOFAS (American Orthopaedic Foot & Ankle Society) ankle-hindfoot scale,[23] SF-36 PCS (Short Form-36 Physical Component Summary) score,[24] and VAS (visual analog scale) for pain. Outcomes were assessed by blinded observers not involved in the procedures. Postoperative complications, such as surgical site infection and wound dehiscence (defined as partial or complete separation of incisions), were monitored.

### Magnetic Resonance Imaging (MRI) evaluation

Preoperative MRI assessed lesion size, location, subchondral cyst presence, and cartilage injury status. OLT size was calculated using the elliptical formula via a picture archiving and communication system (PACS, version 5.4; INFINITT Healthcare): area = maximum coronal length × maximum sagittal length × π/4 .[25]

Postoperative 3.0-T MRI with fat-suppressed, 3-dimensional fast spoiled gradient-recalled acquisition in steady state was performed at least 6 months after surgery, with patient consent, to evaluate repaired cartilage. The Magnetic Resonance Observation of Cartilage Repair Tissue (MOCART) 2.0 scoring system (Table 1),[26] consisting of 7 variables (reduced from 9), with scores ranging from 0 to 100 points, was used for assessment.

**Table 1:** MOCART 2.0 Score: Cartilage repair tissue assessment - grading and point scale.

		Score
**Volume fill of cartilage defect**		
1	Complete filling OR minor hypertrophy: 100% to 150% filling of total defect volume	20
2	Major hypertrophy ≥150% OR 75% to 99% filling of total defect volume	15
3	50% to 74% filling of total defect volume	10
4	25% to 49% filling of total defect volume	5
5	<25% filling of total defect volume OR complete delamination in situ	0
**Integration into adjacent cartilage**		
1	Complete integration	15
2	Split-like defect at repair tissue and native cartilage interface ≤2 mm	10
3	Defect at repair tissue and native cartilage interface <2 mm, but <0% of repair tissue length	5
4	Defect at repair tissue and native cartilage interface ≥50% of repair tissue length	0
**Surface of the repair tissue**		
1	Surface intact	10
2	Surface irregular <50% of repair tissue diameter	5
3	Surface irregular ≥50% of repair tissue diameter	0
**Structure of the repair tissue**		
1	Homogeneous	10
2	Inhomogeneous	0
**Signal intensity of the repair tissue**		
1	Normal	15
2	Minor abnormal—minor hyperintense OR minor hypointense	10
3	Severely abnormal—almost fluid like OR close to subchondral plate signal	0
**Bony defect or bony overgrowth**		
1	No bony defect or bony overgrowth	10
2	Bony defect: depth <thickness of adjacent cartilage OR overgrowth <50% of adjacent cartilage	5
3	Bony defect: depth ≥ thickness of adjacent cartilage OR overgrowth ≥50% of adjacent cartilage	0
**Subchondral changes**		
1	No major subchondral changes	20
2	Minor edema-like marrow signal—maximum diameter <50% of repair tissue diameter	15
3	Severe edema-like marrow signal—maximum diameter ≥50% of repair tissue diameter	10
4	Subchondral cyst ≥5 mm in longest diameter OR osteonecrosis-like signal	0

MOCART, Magnetic Resonance Observation of Cartilage Repair Tissue.

MRI findings were evaluated by 2 independent, blinded observers not involved in the procedures. For continuous variables like OLT size, each observer measured the same image twice, and the mean of the values, excluding the extremes, was used. For categorical variables, such as MOCART score subcategories, the less favorable assessment was chosen in case of disagreement.

### Statistical analyses

Descriptive statistics were calculated using standard formulas. The Kolmogorov–Smirnov test assessed data normality. Group differences were analyzed using the independent t-test for normally distributed data and the Mann-Whitney U test otherwise. Multivariable regression adjusted clinical outcomes for age, sex, BMI, and diabetes mellitus status as potential confounders. Categorical variables were compared using the chi-square test or Fisher’s exact test. Statistical analyses were conducted using SPSS Statistics for Windows, version 23.0 (IBM Corp., Armonk, NY, USA), considering p < 0.05 as statistically significant.

## Results

Table 2 presents the demographic data primary arthroscopic findings for each group. The smoker group’s mean age at surgery was 35.9 years (range, 20–62), while the nonsmoker group’s was 38.8 years (range, 18–65) (p = 0.157). The smoker group had a significantly higher proportion of men (p < 0.001). Smokers had a mean smoking history of 12.0 pack-years (range, 1.0–40.7) at surgery and 20.4 pack-years (range, 18.2–44.4) at the last follow-up. No significant intergroup differences were found in BMI or diabetes prevalence (p = 0.313, p = 0.204). The mean follow-up duration was 83.6 months (range, 24–217), with no significant intergroup difference (p = 0.582).

**Table 2:** Patient demographic characteristics.

	Smoker group	Nonsmoker group	P value^*^
No. of patients (ankles)	56 (59)	183 (191)	
Age^C^ (yr)	35.9 ± 13.1 (20-62)	38.8 ± 15.6 (18-65)	0.157
Sex^‡^			<0.001
Male	58 (98.3%)	108 (56.5%)	
Female	1 (1.7%)	83 (43.5%)	
Body mass index^†^ (kg/m^2^)	25.8 ± 4.4 (18.7-44.4)	25.2 ± 3.9 (18.2-37.0)	0.313
Diabetes^‡^	–	7 (3.7%)	0.204
Pack-Years of Smoking^†^ (yr)	12.0 ± 10.5 (1.0-40.7)	–	
Follow-up duration^†^ (mo)	86.8 ± 55.5 (24-207)	82.6 ± 51.0 (24-217)	0.582
Primary arthroscopic Findings^‡^ (Ferkel and Cheng stage)			0.474
Stage A	2 (3.4%)	4 (2.1%)	
Stage B	6 (10.2%)	23 (12.0%)	
Stage C	5 (8.5%)	33 (17.3%)	
Stage D	29 (49.2%)	80 (41.9%)	
Stage E	12 (20.3%)	41 (21.5%)	
Stage F	5 (8.5%)	10 (5.2%)	

* The independent t-test was used to analyze differences in age, body mass index, and follow-up period. The chi-square or Fisher’s exact test was used to analyze differences in sex and diabetes status. A p-value < 0.05 was considered significant.

† The values are given as the mean ± standard deviation, with the range in parentheses.

‡ The values are given as the number of ankles, with the percentage in parentheses.

In terms of primary arthroscopy, it revealed F–C stage D as the most common cartilage injury status, with approximately 80% of patients in both groups presenting with F–C stage C, D, or E injuries. There was no significant difference in F-C stage in primary arthroscopy between the two groups (p = 0.474).

### Clinical outcomes

All primary and secondary clinical outcome variables improved from preoperative to last follow-up scores in the two groups (Table 3). In primary clinical outcome, there was no significant difference in all FAOS subscale between smoker group and nonsmoker group at the final follow-up after adjusting for baseline characteristics (p > 0.05). In secondary clinical outcomes, both groups achieved comparable results in AOFAS ankle-hindfoot scores, SF-36 PCS scores, and VAS pain scores at the last follow-up (p > 0.05). However, there was no significant difference in the improvement of each FAOS subscale between the two groups, while the nonsmoker group showed significantly greater improvement in AOFAS ankle-hindfoot scores, SF-36 PCS scores, and VAS for pain (p < 0.05). No postoperative complications, including surgical site infection and wound dehiscence, were reported in either group.

**Table 3:** Comparison of clinical outcomes between the smoker group and nonsmoker group following microfracture for OLT.

	Smoker Group (59 ankles)	Nonsmoker Group (191 ankles)	P Value^*^	
			Independent t-test	Multivariable Analysis
FAOS pain^†^				
Preoperative	64.9 ± 13.9 (16.7-94.4)	62.9 ± 11.5 (19.4-97.2)	0.329	0.360
Final	84.3 ± 12.9 (47.2-100)	84.8 ± 10.7 (30.6-100)	0.742	0.393
Improvement	19.4 ± 14.5	22.0 ± 19.0	0.342	0.054
FAOS symptoms^†^				
Preoperative	62.0 ± 14.7 (10.7-92.9)	62.8 ± 14.0 (21.4-92.9)	0.716	0.472
Final	85.2 ± 14.7 (25.0-100)	82.8 ± 13.0 (35.7-100)	0.231	0.286
Improvement	23.3 ± 15.9	20.0 ± 16.6	0.188	0.935
FAOS ADL^†^				
Preoperative	75.2 ± 14.0 (11.8-97.1)	75.1 ± 12.7 (16.2-97.7)	0.992	0.774
Final	92.5 ± 8.4 (63.2-100)	91.2 ± 8.0 (47.1-100)	0.275	0.882
Improvement	17.3 ± 13.9	16.2 ± 13.8	0.577	0.712
FAOS Sport/Rec^†^				
Preoperative	52.5 ± 15.2 (5.0-90.0)	51.4 ± 15.2 (5.0-85.0)	0.655	0.697
Final	78.7 ± 17.5 (20.0-100)	77.9 ± 14.2 (10.0-100)	0.748	0.474
Improvement	26.0 ± 16.0	26.5 ± 21.6	0.874	0.202
FAOS QOL^†^				
Preoperative	47.2 ± 12.6 (12.5-81.3)	46.6 ± 13.5 (6.3-87.5)	0.753	0.972
Final	70.9 ± 18.0 (12.5-100)	70.8 ± 15.9 (6.3-100)	0.977	0.624
Improvement	23.7 ± 21.1	24.3 ± 20.6	0.857	0.442
AOFAS ankle-hindfoot score^†^				
Preoperative	73.0 ± 8.2 (52.0-87.0)	72.2 ± 6.7 (53.0-87.0)	0.512	0.662
Final	88.3 ± 10.6 (56.0-100)	90.8 ± 7.4 (63.0-100)	0.124	0.145
Improvement	15.3 ± 10.8	18.6 ± 9.3	0.024	0.008
SF-36 PCS score^†^				
Preoperative	59.7 ± 13.1 (25.0-90.5)	57.0 ± 12.5 (25.0-94.0)	0.171	0.167
Final	74.3 ± 11.8 (34.0-95.0)	76.2 ± 13.7 (16.9-100)	0.355	0.136
Improvement	14.7 ± 14.6	19.2 ± 17.0	0.046	0.019
VAS for pain^†^				
Preoperative	5.1 ± 1.6 (1.0-7.0)	5.3 ± 1.3 (1.0-8.0)	0.291	0.425
Final	2.2 ± 1.8 (0.0-7.0)	1.8 ± 1.4 (0.0-7.0)	0.115	0.144
Improvement	2.9 ± 2.0	3.6 ± 1.7	0.012	0.004

* The independent t-test was used to analyze differences in FAOS pain, FAOS symptoms, FAOS ADL, FAOS Sport/Rec, FAOS QOL, AOFAS ankle-hindfoot, SF-36 PCS, and VAS for pain scores. Multivariable regression was used to adjust for baseline characteristics, including age, sex, body mass index, diabetes mellitus status, lesion size, and primary arthroscopic Ferkel and Cheng stage as potential confounders. A p-value < 0.05 was considered significant.

† The values are given as the mean ± standard deviation, with the range in parentheses.

OLT, osteochondral lesions of the talus; FAOS, Foot and Ankle Outcome Score; ADL, activities of daily living; Sport/Rec, sport and recreation function; QOL, quality of life; AOFAS, American Orthopaedic Foot & Ankle Society; SF-36 PCS, Short Form-36 Physical Component Summary; VAS, visual analog scale.

### MRI outcomes

MRI findings are detailed in Table 4. Preoperative assessments revealed no significant difference in lesion size between the groups (p = 0.322). Most lesions (204 ankles, 81.6%) were located medially, with only 2 central lesions in the nonsmoker group. Smokers had a higher incidence of subchondral cysts preoperatively (p = 0.002).

**Table 4:** Comparison of MRI outcomes and arthroscopic findings between the smoker group and nonsmoker group following microfracture for OLT.

	Smoker Group (59 ankles)	Nonsmoker Group (191 ankles)	P value^*^
**Preoperative MRI**	**N = 59**	**N = 191**	
Lesion size^†^ (mm^2^)	74.4 ± 27.2 (26.3–145.9)	69.9 ± 30.1 (20.1–148.3)	0.322
Location of lesion^‡^			0.852
Medial	47 (79.7%)	157 (82.2%)	
Central	–	2 (1.0%)	
Lateral	10 (16.9%)	27 (14.1%)	
Medial & Lateral	2 (3.4%)	5 (2.6%)	
Subchondral cyst^‡^	40 (67.8%)	86 (45.0%)	0.002
**Postoperative MRI**	**N = 18**	**N = 57**	
Bone marrow edema^‡^	12 (20.3%)	30 (15.7%)	0.366
MOCART score^†^ (point)			
Volume fill of cartilage defect (20)	15.0 ± 5.4 (5.0–20.0)	16.0 ± 4.0 (5.0–20.0)	0.599
Integration into adjacent cartilage (15)	11.4 ± 4.5 (0.0–20.0)	11.0 ± 3.5 (5.0–15.0)	0.650
Surface of the repair tissue (10)	3.9 ± 2.7 (0.0–10.0)	5.7 ± 2.8 (0.0–10.0)	0.019
Structure of the repair tissue (10)	6.1 ± 5.0 (0.0–10.0)	7.6 ± 4.3 (0.0–10.0)	0.211
Signal intensity of the repair tissue (15)	10.0 ± 2.4 (5.0–15.0)	11.9 ± 2.5 (10.0–15.0)	0.008
Bony defect or bony overgrowth (10)	4.7 ± 4.5 (0.0–10.0)	6.3 ± 3.5 (0.0–10.0)	0.200
Subchondral changes (20)	13.9 ± 6.3 (0.0–20.0)	15.2 ± 5.3 (0.0–20.0)	0.473
Total (100)	65.0 ± 14.0 (30.0–85.0)	73.7 ± 14.1 (40.0–95.0)	0.027

* The independent t-test was used to analyze differences in lesion size, and the Mann-Whitney U test was used to analyze the difference in mean MOCART scores. The chi-square or Fisher’s exact test was used to analyze differences in lesion location, subchondral cyst presence, and bone marrow edema presence. A p-value < 0.05 was considered significant.

† The values are given as the mean ± standard deviation, with the range in parentheses.

‡ The values are given as the number of ankles, with the percentage in parentheses.

MRI, magnetic resonance imaging; OLT, osteochondral lesions of the talus; MOCART, Magnetic Resonance Observation of Cartilage Repair Tissue.

**Fig. 1:**
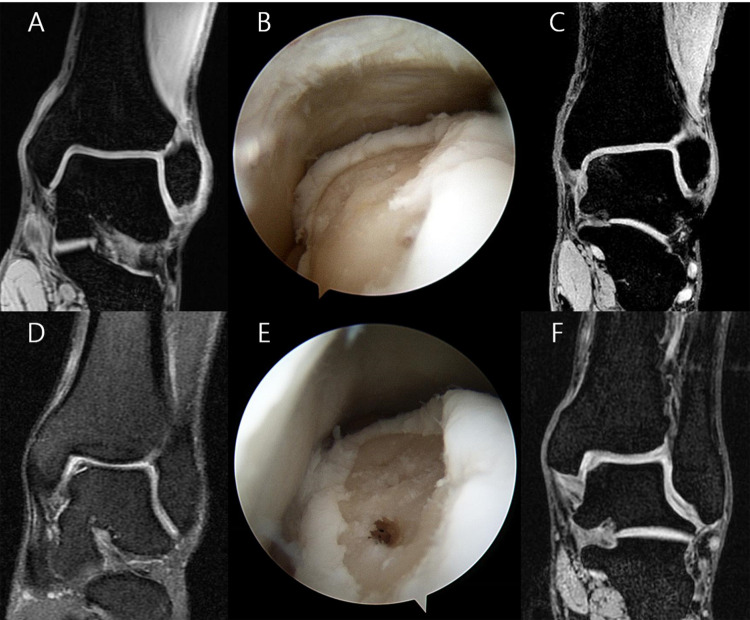
(A) In a 53-year-old male smoker, a preoperative coronal T2-weighted MRI shows an osteochondral lesion of the talus with fibrillated cartilage and no evidence of subchondral bone edema. (B) An arthroscopic image taken after microfracture treatment corresponds to Ferkel and Cheng stage D. (C) A follow-up T2-weighted MRI performed 3 years postoperatively reveals a minor hypointense signal and split-like defect at the repaired cartilage with mild subchondral edema (MOCART score 70 points). (D) In a 21-year-old male non-smoker, a preoperative coronal T2-weighted MRI reveals an osteochondral lesion on the talus with a fully detached but non-displaced fragment. (E) An arthroscopic image taken following microfracture treatment, classified as Ferkel and Cheng stage E. (F) A follow-up T2-weighted MRI performed 2.7 years postoperatively shows the defect completely filled with repair tissue and full integration with adjacent cartilage without subchondral change (MOCART score 85 points).

Postoperative MRI was performed on 75 of the 250 ankles (18 smokers and 57 nonsmokers) to assess the status of repaired cartilage (Fig 1). The mean follow-up duration from surgery to MRI was 44.6 months in the smoker group and 36.1 months in the nonsmoker group, with no significant difference between the two groups (p = 0.196). Additionally, there were no significant differences in baseline characteristics between the two groups, including age, BMI, and diabetes prevalence (p > 0.05). Subchondral bone marrow edema was present in 12 ankles (20.3%) of the smoker group and 30 ankles (15.7%) of the nonsmoker group (p = 0.366). The smoker group had significantly lower MOCART scores for repair tissue surface and signal intensity (p = 0.019, p = 0.008). The mean total MOCART score was 65.0 (range, 30.0–85.0) in smokers, significantly less than the nonsmoker mean of 73.7 (range, 40.0–95.0) (p = 0.027).

## Discussion

The negative effects of smoking on surgical outcomes are well-documented in association with orthopedic procedures, including arthroplasty, spine, fracture, and foot and ankle procedures.[27–33] However, to our knowledge, the specific effects of smoking on arthroscopic microfracture outcomes for OLT have not been previously reported. This study was first study in comparing the clinical outcomes of microfracture for OLT in smokers with those of nonsmokers. Its strength lay in evaluation of consecutive cases and the assessment of repaired cartilage via postoperative MRI. The smoker group exhibited comparable clinical outcomes compared with the nonsmoker group, a result that persisted even after adjusting for baseline characteristics among patients. However, the smoker group had notably poorer MOCART scores in postoperative MRI.

The exact pathophysiological mechanisms by which smoking affects articular cartilage repair remain unclear. Articular cartilage relies on diffusion from synovial fluid and subchondral bone for oxygen and nutrient uptake rather than direct blood supply.[34] Nicotine impairs vascular endothelial function and enhances platelet aggregation, potentially leading to microvascular occlusion.[35] It also induces vasoconstriction, exacerbating local tissue ischemia. Additionally, carbon monoxide diminishes hemoglobin’s oxygen-carrying capacity, and hydrogen cyanide intensifies ischemia by inhibiting cellular enzymes.[36] Recent research has also indicated that smoking decreases synovial cytokine expression associated with cartilage metabolism in knee joints.[37]

The relationship between smoking and cartilage healing remains a subject of debate. Jaiswal et al[18] conducted a case-control study on the outcomes of ACI for full-thickness chondral defects of the knee, finding that smokers benefited less from surgical procedures than nonsmokers and identified smoking as a primary risk factor for graft failure. Conversely, Betz et al.[38] observed no differences in clinical outcomes among ex-smokers, smokers, and nonsmokers within the first 2 years after third-generation ACI. Kanneganti et al[39] reviewed 6 studies to explore the biomechanical and clinical relationship between smoking and articular cartilage, suggesting a negative influence, though the association remains unclear.

Specific research on the impact of smoking on OLT cartilage repair is scarce, with—to our knowledge—only 2 case series on AMIC available. Viehöfer et al[17] evaluated 35 AMIC patients and identified smoking as the sole factor associated with an increased need for additional surgery. Smoking status also tended to correlate with poorer postoperative clinical outcomes, although not reaching statistical significance (p = 0.054). Waltenspül et al[13] found that smoking significantly increased the risk of revision surgery for AMIC-related complications (odds ratio 3.7, p = 0.019). However, these studies focused on risk factors and complications rather than conducting a comparative analysis of clinical outcomes.

In our comparative study, no significant difference in final clinical outcomes was observed between smokers and nonsmokers. Interestingly, the MOCART scores were lower in the smoker group for most subscales, as well as the total score, indicating potentially inferior biomechanical properties of the articular cartilage.[40] Prior research has also revealed that nicotine reduces cytokine expression and levels of extracellular cartilage matrix proteins like type I and II collagen in animal models.[37] Thus, we speculate that the structurally inferior repaired cartilage in smokers may impact long-term clinical outcomes due to durability concerns. However, we did not identify inferior final clinical outcome that could be caused by a worse MOCART score in smoker group. Furthermore, when analyzing only the patients who underwent postoperative MRI, no significant differences were found in any FAOS subscale between the two groups in all clinical outcome variables (Table 5). Additionally, the smoker group exhibited a higher incidence of preoperative subchondral cysts, which are typically associated with high fluid pressure on exposed subchondral bone.[41] Another study suggested that subchondral ischemia might contribute to cyst formation,[42] leading us to speculate that smoking could exacerbate cyst development. The MOCART scoring system includes subchondral changes as a variable. Although there was no statistically significant difference in subchondral changes between the two groups (p = 0.473), the higher prevalence of subchondral cysts in smokers may still have contributed to their lower total MOCART scores. In this study, we did not find that lower MOCART scores directly translate into worse clinical outcomes. Further studies with extended follow-up are needed to determine the long-term implications of differences in MOCART scores.

**Table 5:** Comparison of final clinical outcomes between smokers and nonsmokers who underwent postoperative MRI following microfracture for OLT.

	Smoker Group (18 ankles)	Nonsmoker Group (57 ankles)	P value^*^
FAOS pain^†^	77.7 ± 17.3	85.4 ± 12.9	0.095
FAOS symptoms^†^	81.0 ± 15.8	83.3 ± 13.2	0.534
FAOS ADL^†^	90.8 ± 10.8	91.7 ± 9.2	0.725
FAOS Sport/Rec^†^	77.2 ± 20.8	78.3 ± 16.8	0.817
FAOS QOL^†^	65.7 ± 19.6	70.3 ± 18.0	0.357
AOFAS ankle-hindfoot score^†^	83.4 ± 14.2	89.5 ± 8.9	0.103
SF-36 PCS score^†^	72.2 ± 16.0	77.4 ± 12.2	0.148
VAS for pain^†^	3.0 ± 2.3	2.0 ± 1.5	0.098

* The independent t-test was used to analyze differences in FAOS pain, FAOS symptoms, FAOS ADL, FAOS Sport/Rec, FAOS QOL, AOFAS ankle-hindfoot, SF-36 PCS, and VAS for pain scores. A p-value < 0.05 was considered significant.

† The values are given as the mean ± standard deviation.

OLT, osteochondral lesions of the talus; FAOS, Foot and Ankle Outcome Score; ADL, activities of daily living; Sport/Rec, sport and recreation function; QOL, quality of life; AOFAS, American Orthopaedic Foot & Ankle Society; SF-36 PCS, Short Form-36 Physical Component Summary; VAS, visual analog scale.

This study had several limitations. Firstly, the smaller number of smokers compared to the nonsmoker group may have limited the statistical power of our findings. While we aimed to include all eligible patients from a long-term cohort, the lower proportion of smokers reflects the typical demographic distribution of patients undergoing this procedure. Additionally, the number of patients who underwent postoperative MRI was limited, further reducing the sample size available for cartilage repair assessment. Although this remains one of the largest MRI-based studies investigating the effect of smoking on cartilage healing in OLT patients, we acknowledge that the sample size may not be sufficient to draw definitive conclusions. Furthermore, the influence of containment status on outcomes could not be thoroughly analyzed due to the low incidence of contained lesions in both groups, limiting statistical feasibility. Future studies with larger cohorts and multi-center collaboration are needed to enhance generalizability. Secondly, despite adjusting for baseline characteristics through multivariable regression analysis, inherent differences between smokers and nonsmokers—such as gender distribution and potential lifestyle variations—may have influenced the results. Thirdly, given the long study period, variations in rehabilitation protocols may exist. Even though we have made efforts to maintain a consistent postoperative rehabilitation protocol throughout the study period, such variations could have influenced clinical and imaging outcomes. Finally, the study did not include histological assessments of the repaired cartilage tissue to evaluate biomechanical properties.

## Conclusion

This study demonstrated that smokers achieved comparable clinical outcomes after arthroscopic microfracture for small to medium-sized OLT over a mean follow-up of 7 years. While postoperative MRI findings showed that the smoker group had worse MOCART scores regarding repaired cartilage status, these results did not appear to significantly impact clinical outcomes. This discrepancy may be attributed to the higher prevalence of subchondral cysts in the smoker group and the fact that not every patient in each group underwent postoperative MRI assessment. Future larger-scale and longer-term studies are warranted to further elucidate the relationships among smoking, cartilage repair following microfracture, and the clinical implications of lower MOCART scores.

## Supporting information

S1 File Raw data used in this study. (XLSM)
